# Patients’ and GPs’ views and expectations of home monitoring with a pulse oximeter: a mixed-methods process evaluation of a pilot randomised controlled trial

**DOI:** 10.3399/BJGP.2023.0139

**Published:** 2023-11-28

**Authors:** Karin Smit, Roderick P Venekamp, Geert-Jan Geersing, Frans H Rutten, Lisette Schoonhoven, Dorien LM Zwart

**Affiliations:** Department of General Practice, Julius Center for Health Sciences and Primary Care, University Medical Center Utrecht, Utrecht.; Department of General Practice, Julius Center for Health Sciences and Primary Care, University Medical Center Utrecht, Utrecht.; Department of General Practice, Julius Center for Health Sciences and Primary Care, University Medical Center Utrecht, Utrecht.; Department of General Practice, Julius Center for Health Sciences and Primary Care, University Medical Center Utrecht, Utrecht.; Department of General Practice, Julius Center for Health Sciences and Primary Care, University Medical Center Utrecht, Utrecht.; Department of General Practice, Julius Center for Health Sciences and Primary Care, University Medical Center Utrecht, Utrecht.

**Keywords:** COVID-19, general practice, oximetry, pulse oximetry, SARS-CoV-2

## Abstract

**Background:**

Research on how home monitoring with a pulse oximeter is executed and experienced by patients with an acute illness such as COVID-19 and their GPs is scarce.

**Aim:**

To examine the process of structured home monitoring with a pulse oximeter for patients with COVID-19, their caregivers, and their GPs.

**Design and setting:**

This was a mixed-method process evaluation alongside a pilot feasibility randomised controlled trial. Patients drawn from a general practice setting, with COVID-19, and aged ≥40 years with cardiovascular comorbidities were included.

**Method:**

Quantitative trial data from 21 intervention group participants (age 63.2 years) were used, plus qualitative data from semi-structured interviews with 15 patients (age 62.9 years), eight informal caregivers, and 10 GPs.

**Results:**

Adherence to the intervention was very high; 97.6% of protocolised peripheral oxygen saturation (SpO_2_) measurements in the first 14 days until admission to hospital were recorded (677/694, median daily per patient 2.7). Three identified themes from the interviews were: (a) user-friendliness of home monitoring: easy use of the pulse oximeter and patient preference of a three times daily measurement scheme; (b) patient empowerment: pulse oximeter use enhanced patient self-assurance and empowered patients and informal caregivers in disease management; and (c) added value to current clinical decision making. GPs perceived the pulse oximeter as a useful diagnostic tool and did not experience any additional workload. They felt more secure with remote monitoring with a pulse oximeter than only phone-based monitoring, but emphasised the need to keep an overall view on the patient’s condition.

**Conclusion:**

Structured home monitoring by pulse oximetry supports patients and their informal caregivers in managing, and GPs in monitoring, acute COVID-19 disease. It appears suitable for use in acutely ill patients in general practice.

## INTRODUCTION

The role of pulse oximetry as a home monitoring tool has become increasingly popular since the start of the COVID-19 era. As a result of overwhelmed healthcare systems, home monitoring initiatives were pragmatically implemented with the overarching hypothesis that self-monitoring with a pulse oximeter could potentially detect progression of COVID-19 and associated (happy or silent) hypoxaemia, warranting intensive treatment in an early stage of the disease.^1^^–^3

In the pre-COVID era, studies on how self-monitoring at home with pulse oximetry is perceived and experienced by patients has mainly been conducted with patients who have chronic diseases such as asthma and chronic obstructive pulmonary disease (COPD).^4^^,^^5^ For these conditions, patients (for example, those with COPD) reported mixed experiences; some patients felt safe, but others felt less safe or even anxious.^6^^,^^7^

During an acute illness, patients’ views and expectations about home monitoring, as well as their GPs, may differ from that of patients with a chronic illness because, in general, acute symptoms necessitate monitoring only for a relative short period. Typically, patients who are acutely ill act and feel different, because of the sudden occurrence of mostly unfamiliar, and sometimes threatening, symptoms, for example, shortness of breath.^1^^,^^8^^,^^9^

A previous Australian qualitative study focused on remote monitoring and use of pulse oximetry among patients with COVID-19 who received on average 8 days of home monitoring after emergency department consultation; the study reported positive perceptions from both patients and healthcare providers.^9^

Also, a scoping review on user perceptions about both hospital and remote monitoring programmes reported positive perceptions from both patients and healthcare providers. This was among patients with COPD, cardiovascular disease, and children with pneumonia.^10^ In addition, in a scoping review in eight countries of patients with acute respiratory infection during the first wave of COVID-19, and their healthcare providers, remote consultations were accepted by patients, but GPs thought it negatively affected the doctor–patient relationship.^11^

In the current study, the aim was therefore to answer the following questions:

- did patients with COVID-19 use the pulse oximeter as instructed during home monitoring?; and
- what are the views and expectations of patients with COVID-19, their informal caregivers, and involved GPs about structured home monitoring with a pulse oximeter?

**Table:** How this fits in

Home monitoring with a pulse oximeter by patients with an acute illness such as COVID-19 has become increasingly popular. Patients, informal caregivers, and GPs perceive the pulse oximeter as user-friendly. Its use enhanced patients’ self-assurance and empowers patients and informal caregivers in disease management, and supports GPs in monitoring acute COVID-19 disease.

## METHOD

### Study design

Following the Medical Research Council guidance for process evaluation of complex interventions,^12^ a mixed-methods process evaluation was conducted alongside CovidSat@Home, a pilot randomised controlled feasibility trial, which has been published previously about the use of a validated pulse oximeter for monitoring patients with COVID-19 at home.^13^ A sequential explanatory design was used, in which the quantitative trial data was collected and analysed first. Subsequently, patients, informal caregivers, and the involved GPs were interviewed. The main goal of the interviews was to enrich and interpret the quantitative data.^14^^–^16

Quantitative trial data were used to examine how the intervention was used in practice in terms of:

- fidelity (the extent to which an intervention is delivered as intended);
- dose (the quantity of what is delivered);
- adaptations (alterations made to an intervention to achieve better contextual fit); and
- reach (the extent to which a target audience comes into contact with the intervention).

In this study, fidelity was operationalised as the actual delivery of the intervention by the researcher to the patient as intended in the protocol. The outcome ‘dose’, operationalised as the extent of the participant’s engagement in the use of the intervention, was measured using data from patient-reported peripheral oxygen saturation (SpO_2_) readings during the study period collected by patients in a paper diary. In case adaptations to the intervention were made during the study period, these were reported in a logbook by the researchers. For the outcome ‘reach’ the number of participants who actually participated in applying the intervention was assessed. This data were acquired during the intervention (the first 14 days).

Between day 14 and 28 after the start of the intervention, semi-structured interviews with patients, their informal caregivers, and involved GPs were undertaken to collect data on their experiences and perceptions about the use of a pulse oximeter as a home monitoring tool. A conventional content analysis approach was used with coding that derived inductively from the research data.^17^

### Brief description of CovidSat@Home and the intervention

The results of the pilot randomised controlled trial (RCT) CovidSat@Home have been published previously.^13^ In brief, a primary care-based, open-label, individually randomised, pilot RCT was conducted to assess the feasibility of a trial on home monitoring by pulse oximetry for patients aged ≥40 years with cardiovascular comorbidity and moderate-to-severe COVID-19 disease.

From December 2020 to June 2021, eligible patients were randomly allocated to regular measurement of SpO_2_ with a validated pulse oximeter or to usual primary care. Patients in the intervention group received instructions on how to use the pulse oximeter. This was accompanied by standardised written, verbal, and visual information. By protocol, measurements should be performed three times a day for 14 days plus additional measurements after 5 min of rest if SpO_2_ was <94%. In the case of persisting hypoxaemia, patients were instructed to contact their GP. Patients were also instructed to contact their GP in the case of clinical deterioration, irrespective of SpO_2_ levels. Patients in the intervention group recorded their spot SpO_2_ measurements in a paper diary. All patients (intervention *n* = 21, control *n* = 20) completed a 45-day follow-up period.^9^

### Semi-structured interviews

#### Sampling and recruitment

All patients from the trials’ intervention group who consented to be interviewed (18/21) were consecutively sampled for this process evaluation study. After completion of the 14-day monitoring period, an interview was planned. In total, eight informal caregivers were interviewed along with 15 patients.

All 10 GPs involved in the care of the patients in the intervention group of the pilot RCT were asked to participate in an interview. After verbal informed consent was given by all 10 GPs, interviews were planned.

#### Topic list

The interview topic lists for both patients and GPs were developed based on a literature review and expert opinion.^5^^,^^18^^,^^19^ Two main topics were discussed:

- the use of the pulse oximeter as a home monitoring device; and
- how perceptions and experiences with its use had an effect on the participants’ perception of safety regarding COVID- 19.

The authors planned to further shape the topic guide during the interviews if new topics arose. Ultimately, only one item was added; this was related to the role of the informal caregiver (if present) during the monitoring process (see Supplementary Table S1 for the topic list for patient interviews and Supplementary Table S2 for the topic list for the GP interviews).

#### Interviews

Twenty-five semi-structured interviews were conducted with 15 patients along with their eight informal caregivers and with 10 GPs between day 14 and day 28 after trial enrolment. The interviews took place at the patient’s home or GP practices and were conducted by the first author and two final-year medical residents after training and under supervision of the first author. The researchers had no personal or professional relationship with the participants. The interviews were audio-recorded and directly uploaded to the University Medical Center Utrecht research folder, which could only be accessed by assigned researchers of the research group. The audio was then pseudonymised and transcribed verbatim. Transcripts were not returned to participants for reading or comment.

### Analysis

The quantitative data were descriptively analysed using SPSS Statistics version 26.

Qualitative data were analysed by thematic analysis where the main interview topics were used as a starting point for the formation of themes. The transcripts were first systematically read and independently coded by two researchers (the first and fifth author) and two final-year medical residents. Discrepancies in coding were discussed until consensus was reached and themes were formed by summarising the codes (the coding tree is provided in Supplementary Table S3). Data were structured and analysed using NVivo (version 12). Data collection and analysis occurred concurrently. Triangulation within the group of researchers was used to ensure the quality of coding. When no new themes came up, the authors defined that data saturation was reached. The ‘Consolidated criteria for reporting qualitative studies (COREQ): 32-item checklist’ was used to report the study.^20^

## RESULTS

### Participant characteristics

Of the 21 trial participants allocated to the intervention group, 8 (38.1%) were female and the mean age was 63.2 years (Table 1).

**Table 1.** Patient characteristics of the intervention group participants

**Characteristic**	**Intervention group (*n* = 21)**
**Female sex, *n* (%)**	8 (38.1)
**Age, years, mean (SD)**	63.2 (10)
**Hospital admissions, *n* (%)**	5 (23.8)

*SD = standard deviation.*

Fifteen patients (71.4%) were interviewed. Reasons for not participating in an interview were: no consent given (*n* = 3), being too busy (*n* = 1), could not be reached (*n* = 1), and data saturation was reached (*n* = 1). Almost all participating patients (14/15, 93.3%) lived together with an informal caregiver. Of those, eight were present during the interviews and were actively involved in part of the interview. The interviews lasted on average 33 min (standard deviation [SD] 9). Characteristics of the interview patients are given in Table 2.

**Table 2.** Characteristics of the interviewed patients (*n* = 15)

**Characteristic**	**Interviewed patients (*n* = 15)**
**Female sex, *n* (%)**	4 (26.7)

**Age, years, mean (SD)**	62.9 (8.9)

**Medical history, *n* (%)**	
Hypertension	8 (53.3)
Coronary artery disease	1 (6.7)
Hypercholesterolaemia	9 (60.0)
Chronic lung disease	2 (13.3)
Diabetes mellitus	4 (26.7)
Stroke or TIA	4 (26.7)

**Living with informal caregiver, *n* (%)**	14 (93.3)

**Hospital admission during 45-day follow-up, *n* (%)**	4 (26.7)

*SD = standard deviation. TIA = transient ischaemic attack.*

The 10 participating GPs (six males) worked in eight different practices in the vicinity of Utrecht and Amsterdam. The interviews with the GPs lasted on average 18 min (SD 6).

### Fidelity, dose, adaptations, and reach: did patients with COVID-19 use the pulse oximeter for structured home monitoring as instructed?

All 21 patients in the intervention group received a validated (for medical use) pulse oximeter according to protocol and performed the first measurement in the presence of the researcher (100% fidelity). All completed the 45-day follow-up period and no patients withdrew from the study.

All patients performed at least one SpO_2_ measurement after study inclusion (100% reach). A total of 727 SpO_2_ measurements (median daily measurements per patients 2.7; interquartile range 1–4) were recorded in the paper diaries in the first 14 days (97.6% [677/694] of protocolised measurements) until hospitalisation (dose).

Hypoxaemia (SpO_2_ <94%) was recorded 52 times by 10 patients. Of those patients, six contacted their GP as instructed. From the other four patients, three already had a regular follow-up appointment with their GP planned on the same or next day and therefore did not reach out actively. The last patient that did not contact the GP as instructed measured an SpO_2_ level of 93% for a total of three measurements on day one and two, and deemed it not necessary to call the GP. During the study, no adaptations to the intervention protocol were made, nor needed to be made. Further quantitative trial data about patients’ characteristics and the outcomes have been published previously in the *BJGP*.^13^

### Views and expectations of structured home monitoring with a pulse oximeter of patients, their informal caregivers, and GPs

Three main themes were formed after analysing the interview data:

- user-friendliness of home monitoring;
- patient empowerment; and
- added value to current clinical decision making.

#### User-friendliness of home monitoring

All patients, informal caregivers, and GPs found the pulse oximeter easy to use in terms of functionality, application, and reading of the measurements; this was convergent across all groups. For patients, only one patient mentioned that they had used the written instructions and no one watched the instructional video. In fact, none of the patients or informal caregivers needed external help about using the device or reading the measurements from the pulse oximeter screen:

*‘ Yes, was pretty easy. They made it very easy indeed. You put it on and it works […] I didn’t watch the movie clip. I actually found it straightforward.’*

(Patient C032, 62 years)

Patients reported that they usually did perform SpO_2_ measurements three times daily as instructed. The exact times at which they performed measurements differed, but they all mentioned measuring in the morning after rising, around lunchtime, and after the evening meal. Patients found the use of the paper diary helpful as a reminder; they often stored this together with the pulse oximeter in a central place in the house, for example, on the kitchen table, and so it served as a visual memory aid:

*‘ We just did that three times a day, during the meals. With breakfast in the morning, with lunch and dinner, sure, that’s easy to remind* [you]*.’*

(Patient C037, 62 years)

As a critical note, some patients mentioned that the instructed measurement period of 14 consecutive days was a bit too extensive. This was when measured oxygen saturation levels remained the same (no variability) and it was considered inconvenient taking the pulse oximeter with them when leaving the house after the COVID-19 quarantine period was over:

*‘ I mean, I leave that monitor here* [at home*]. I’m not going to take it anywhere, everywhere, with me.’*

(Patient C012, 65 years)

#### Patient empowerment

This theme was derived specifically from patient and informal caregiver data. When asked about feelings of anxiety, patients mentioned the influence of media covering COVID-19 items.

These media items caused increased feelings of fear about developing serious illness and admission to the intensive care unit for mechanical ventilation support. The need to feel more secure was a reason for patients to participate and actually use the pulse oximeter.

Also, during the course of the disease, the pulse oximeter was used by the patients to gain greater control over their condition by lowering feelings of uncertainty and fear:

*‘ Well, I was just scared. I think: right, just if I … You watch all those images on TV and everything. And of intensive care and hospitals and all those situations. I think: well, I really shouldn’t go that way. But well, when I was really that ill, and I’m actually never ill, so well then, I really had the impression that well, I have to take care at least that I do not get even more breathless, because then it will go wrong.’*

(Patient C012, 65 years)

Patients indicated that both the prescribed structured measurements (three times daily for 14 days) and extra measurement times were checks if they were ‘okay’. This was especially important at the beginning of the acute illness period as participants felt the need to reassure themselves about still being ‘okay’, indicating they had a normal oxygen saturation level, and thereby feel more secure. After patients went into a recovery phase, they used the pulse oximeter to check whether their oxygen levels matched their impression of the improvement in their clinical condition. By doing this, they felt more assured they indeed were recovering:

*‘ If you are above 96, 97 per cent, then you know you are okay. Then you are safe, or how you want to call it.’*

(Patient C024, 70 years)

*‘ And on a certain moment, after a couple of weeks you see an increase and above 93, 94, 95 per cent … well, then you just are happy. Then you think: Okay, we’re going in the right direction.’*

(Patient C033, 57 years)

If patients felt they were ‘not okay’ (indicating a low oxygen saturation level or worsening clinical condition), the pulse oximeter provided guidance on whether or not they needed to take action and call the GP. The pulse oximeter was often compared with a thermometer as an extra tool to objectify their feelings of ‘not being okay’. With this extra information, patients felt empowered to call a healthcare professional if necessary:

*‘Well yes, you had a reason to call the GP. And what the GP would do with it, yes that’s up to him. Look, because if I wouldn’t* [have] *had this thing, this device, then I wouldn’t know if I had 92 or 96. And I think that if you don’t know it, then they will only find it out when you start creaking and squeaking. But well, that didn’t happen to me. So … But that could be a reason to call your GP, yes.’*

(Patient C022, 50 years)

As indicated above, patients felt empowered during the course of the disease by pulse oximetry. There were no negative experiences mentioned, such as increased anxiety or experiencing barriers when contacting a GP.

Informal caregivers indicated that this feeling of greater control over decisions and actions during the COVID-19 disease course extended to them. This was especially true during clinical deterioration with hypoxaemia. Here, the patients themselves felt confused or not mentally clear, and found it difficult to report and interpret oxygen saturation levels, and what actions to take about these levels. Instead, their informal caregiver had to monitor oxygen levels and interpret these values. Informal caregivers were also the ones to call the GP and they mentioned that they felt empowered to do so because of the additional information the oxygen saturation reading had given them, as an extra argument to ask for help:

*‘ The fact he was really confused … and I was already sitting straight up the entire night with the lights on, so I could keep a good eye on him.’*

(Informal caregiver C024, 70 years)

Four patients did not call in immediate help when confronted with a lower than normal SpO_2_ reading. In light of self-management, patients postponed the call when a regular follow-up visit was already scheduled that day, as the oxygen saturation levels were going to be discussed then. One patient did not call at all as he decided, together with his informal caregiver, that there were very few accompanying symptoms and therefore they trusted their own interpretation that his condition was okay:

*‘ I did know it was low and everything went so quickly that day and really nothing got through to me.’*

(Patient C009, 63 years)

#### Added value to current clinical decision making

This theme was derived from GP data. The pulse oximeter was also perceived as very user-friendly by the GPs and no difficulties were found in instructing patients on how to use the pulse oximeter. When asked about the prescribed frequency, that is, three times daily for 14 days, there was variation in the instructions about frequency of taking measurements that the different GPs would give outside this study context. Only one GP normally instructs patients specifically to measure three times daily, as instructed in the current study’s protocol. Some GPs thought that it was preferable to measure at least once a day, to better understand the situation and measure more often when the patient experienced more symptoms:

*‘And I would look at the reference value. I would say to the patient: “look at what a normal value is when you don’t have complaints. So you know a pattern, that you know where you’re coming from.”‘*

(GP, female, 5 years’ experience)

Other GPs stated that they would not give any advice on frequency at all, no minimum or maximum level, as this was perceived as containing a ‘paternalistic component’:

*‘No, I wouldn’t give any instruction* [on monitoring frequency]*. No, I don’t have a paternalistic* component in that. If someone likes to check five times, then that’s fine by me.’

(GP, male, 12 years’ experience)

All the GPs had in common that they would instruct patients to at least perform measurements when they felt ill or experienced progressive symptoms:

*‘ Especially with COVID you see, the course of the disease can be erratic, the patients can deteriorate suddenly and the symptoms don’t always point in the right direction. I think* [pulse oximetry] *has an added value in this.’*

(GP, male, 12 years’ experience)

*‘ You kind of have to, sometimes, tell them like: do not go measure that saturation twenty times a day, but just do it daily and eventually, if you really experience more symptoms, once or twice a day.’*

(GP, male, 12 years’ experience)

*‘ Yes, that they shouldn’t measure it too often. So in principle three times a day, or at a moment that they would really feel short of breath.’*

(GP, male, 12 years’ experience)

The vast majority of GPs felt their workload did not increase when a patient used a pulse oximeter at home for monitoring COVID-19 symptoms. In fact, they did not experience having any extra workload. Only one GP thought that it led to an additional workload for the practice, in terms of extra calls to the doctor’s assistant (Dutch doctor assistants [*doktersassistente*] play an important role in both triage and performing tasks that could be considered similar to the work of a nurse practitioner).

The addition of home pulse oximetry resulted in GPs feeling more secure about monitoring patients at home. Different reasons for this assurance were mentioned. First, GPs used the pulse oximeter as an extra useful diagnostic tool to monitor disease progress. This was especially at the beginning of the illness when it can be used to keep track of the development of any signs of deterioration or silent hypoxaemia. The pulse oximeter helped provide more precise and objective remote monitoring. And, second, knowing that patients could keep track of oxygen saturation levels at home meant that GPs felt more secure since they expected patients to call if there were decreasing SpO_2_ levels (as instructed):

*‘ Yes with COVID, you had patients, especially with the erratic disease course of corona, when you thought “I do not know which direction this goes”. And then* [with a pulse oximeter] *you would just know: it is all right. And somebody can measure and call when it is not going well.’*

(GP, female, 5 years’ experience)

Risk of incorrectly evaluating a patient’s clinical condition when only assessing the oxygen levels was also mentioned by GPs. Several GPs worried about false-negative readings. This situation could arise when the SpO_2_ reading indicates a normal oxygen saturation level, but the patient’s condition is actually worsening. Here, monitoring with a pulse oximeter could give a false sense of security to both the patient and the GP. The GPs considered it still necessary to keep an eye on the patient’s condition and retain an overall perspective:

*‘ So, it can of course also provide a false sense of security, that’s the thing, so if that saturation seems just very good, but the patient is just clinically terribly ill, that’s the thing. Sure, I think that you always everywhere just ought to keep track of the overall picture.’*

(GP, female, 22 years’ experience)

## DISCUSSION

### Summary

In this study, a process evaluation was undertaken, with the aim of evaluating whether patients with COVID-19 used the pulse oximeter as instructed. In addition, the views and expectations of patients with COVID-19, their informal caregivers, and GPs involved with its use were explored. The intervention was adequately delivered to all patients and all used the intervention (fidelity, reach) and the adherence to the intervention was high (dose). All participants found the pulse oximeter user-friendly. Its use enhanced patient self-assurance and empowered both patients and informal caregivers in disease management. GPs perceived that the pulse oximeter added value to their current clinical decision making but they underlined the need to still maintain an overview of the patient’s condition.

### Strengths and limitations

Protocolised implementation of the intervention by a single researcher led to low inter-researcher variability and consistent workflow. The interviews were conducted shortly after the infectious period (within 14–28 days after enrolment) and therefore reduced recall bias. As interviews with both patients and GPs were conducted it was possible to capture a wide range of experiences and perceptions, and to formulate recommendations enriched with experiences and perceptions of several informal caregivers. Ideally, interviews with the informal caregivers would have been conducted without the presence of the patient to get a (more) objective view as these caregivers play a role in home monitoring of patients who are acutely ill. However, there was no indication that the presence of an informal caregiver influenced the patient’s answers negatively but rather contributed to a more in-depth insight into the patient’s disease course.

### Comparison with existing literature

User-friendliness is defined as easy to use, but also easy to understand how something should be used.^21^ The user-friendliness of the pulse oximeter is an important facilitator that was attributed to high adherence, which confirms acceptability. The adherence rate was in line with adherence rates in previous observational studies among patients with COVID-19.^22^^,^^23^ The results of a recently published Australian study, also performed in general practice, showed large overlap with the current study’s results regarding feasibility and acceptance.^9^ The acceptance of pulse oximetry could have been increased by extrinsic motivational factors. One such factor was that patients wanted to feel more in control of their disease. Media coverage about COVID-19 increased their feelings of anxiety and insecurity. The use of a pulse oximeter made patients feel more in control and thereby decreased feelings of anxiety and insecurity.

When patients feel empowered, they are motivated to use an intervention. A recent systematic review on factors influencing the effectiveness of remote patient monitoring interventions in acute care synthesised six theories of intervention success, and enhanced self-management was found to contribute to this success. Part of this success related to receiving direct feedback from monitoring data; participants in the current study experienced this with the pulse oximeter.^24^ Others, who have studied the use of home monitoring tools in chronic disease management, have reported similar supportive findings on the positive effect of their use on patient empowerment.^25^^,^^26^

Furthermore, this empowerment extends to the informal caregivers. In patients where there is clinical deterioration with hypoxaemia, an informal caregiver can help interpret the results and consult a healthcare professional if necessary, taking on a coordinating role. In cases of hypoxaemia, patients often experience tiredness, diminished concentration, and are less able to make adequate decisions.^3^ The coordination role of informal caregivers has also been reported in a recent qualitative study on informal caregivers’ experiences with monitoring patients with heart failure who are at home, where a certain ‘mastering’ was expressed by the caregivers. It was shown that informal caregivers were able to detect changes in measurement values and discuss this with a care professional for medical support.^27^

As a possible barrier to implementation, it was hypothesised that GPs might be hesitant about using the intervention because of the perceived potential extra workload. In this study, GPs did not experience this. A previous prospective observational study reported a 0.04 full-time equivalent workload reduction for each patient receiving only app-based remote patient monitoring versus telephone contact.^28^

During the interviews, GPs mentioned a variety of instructions they would give patients about the frequency of measurements during usual care outside trial participation. In the current study and in other COVID-19 programmes, patients received specific instructions to measure SpO_2_ three times daily at the same time each day.^29^^,^^30^ Reasons why Dutch GPs are not that strict could be:

- GPs fear an increase in workload when instructing a higher frequency;
- a more patient-centred approach is used and shared decision making leads to tailoring frequency to individual patients; and
- in this study it was observed that GPs trusted that patients would use and interpret the SpO_2_ readings correctly and expected them to contact the GP themselves in the case of a deterioration.^31^^–^33

In the current study, only one patient did not discuss an SpO_2_ measurement of 93% with the GP. In their case, the patient and informal caregiver made a decision not to do so because the patient’s condition was not altered. In general, GPs should be aware that there is a possibility that patients may overestimate their ability to self-manage an acute illness.

Although GPs perceive pulse oximetry as a safe and useful diagnostic tool, they mentioned that a stand-alone value of oxygen saturation can give a false sense of security in assessing a patient’s condition. This emphasises the importance of the GP having an overall perspective about the patient’s condition for timely detection of clinical deterioration and to increase patient safety. This overall picture can help both patients and GPs, while searching for a balance between adequate use of self-monitoring and risks of obsession over the results.

### Implications for research and practice

During the study, no adaptations were made to the intervention by the research team. However, the following indications for adaptations have been found based on the patient and GP interviews.

First, the frequency of monitoring day should be based on the patient’s preferences and clinical condition. A too extensive monitoring period leads to a reduction in compliance and patient satisfaction. The authors recommend monitoring at least until the patient’s clinical condition is stable, with oxygen saturation levels in the normal range for at least 24 h. Patients are willing to perform measurements three times daily; patients should be advised to do so at breakfast, lunch, and dinner.

Second, in this study, patients used a paper diary as a memory aid to keep track of monitoring data. Its use enhanced adherence. If, in the future, a digital programme is used, the authors recommend not only sending daily reminders to patients but also ensuring that patients who are not digitally illiterate are not excluded.

Third, the need for a formal or informal caregiver was not tested but seems essential to aid interpretation of measurements and for contacting healthcare professionals, particularly in cases of patients with hypoxaemia and with a high risk of deterioration. Their presence enhances the safety of home monitoring of patients with COVID-19. This finding is also supported by other qualitative research on informal caregivers’ experiences with both chronic and acute diseases.^27^^,^^34^

In conclusion, this study has shown that structured home monitoring by pulse oximetry supports patients and their informal caregivers in managing, and GPs in monitoring, acute COVID-19 disease, and such an approach appears suitable for use with patients who are acutely ill in general practice.

## Data

Datasets are available. Any supplementary data can be requested from the corresponding author.
